# Active and probe-free intracellular rheology via phase-sensitive thermoviscous flows

**DOI:** 10.1093/pnasnexus/pgag190

**Published:** 2026-06-01

**Authors:** Iliya D Stoev, Madison Bolger-Munro, Antonio Minopoli, Susan Wagner, Venkat Raghavan Krishnaswamy, Elena Erben, Kai Weißenbruch, Nicola Maghelli, Martin Bastmeyer, Carl-Philipp Heisenberg, Moritz Kreysing

**Affiliations:** Max Planck Institute of Molecular Cell Biology and Genetics, Dresden 01307, Germany; Institute of Biological and Chemical Systems — Functional Molecular Systems, Karlsruhe Institute of Technology (KIT), Eggenstein-Leopoldshafen 76344, Germany; Institute of Science and Technology Austria, Klosterneuburg 3400, Austria; Department of Neuroscience, Università Cattolica del Sacro Cuore, Rome 00168, Italy; Max Planck Institute of Molecular Cell Biology and Genetics, Dresden 01307, Germany; Institute of Biological and Chemical Systems — Functional Molecular Systems, Karlsruhe Institute of Technology (KIT), Eggenstein-Leopoldshafen 76344, Germany; Max Planck Institute of Molecular Cell Biology and Genetics, Dresden 01307, Germany; Max Planck Institute of Molecular Cell Biology and Genetics, Dresden 01307, Germany; Department of Cell and Developmental Biology, University College London, London WC1E 6BT, United Kingdom; Zoological Institute, Cell and Neurobiology, Karlsruhe Institute of Technology (KIT), Eggenstein-Leopoldshafen 76344, Germany; National Facility for Life Imaging, Human Technopole, Milan 20157, Italy; Zoological Institute, Cell and Neurobiology, Karlsruhe Institute of Technology (KIT), Eggenstein-Leopoldshafen 76344, Germany; Institute of Biological and Chemical Systems — Biological Information Processing, Karlsruhe Institute of Technology (KIT), Eggenstein-Leopoldshafen 76344, Germany; Institute of Science and Technology Austria, Klosterneuburg 3400, Austria; Max Planck Institute of Molecular Cell Biology and Genetics, Dresden 01307, Germany; Institute of Biological and Chemical Systems — Functional Molecular Systems, Karlsruhe Institute of Technology (KIT), Eggenstein-Leopoldshafen 76344, Germany

**Keywords:** cell mechanics, active microrheology, noninvasiveness, thermoviscous flows, FLUCS

## Abstract

Determination of the rheological properties of cells is known to require active measurements, which largely depend on the internalization of mechanical probes. Here, we circumvent this problem via the introduction of Rheological focused light-induced cytoplasmic streaming (Rheo-FLUCS): an active, yet probe-free approach that leverages light-induced flows to access mechanical changes in complex systems. While Rheo-FLUCS is facilitated by thermoviscous expansion phenomena rather than external forces, here we show equivalence in its ability to measure relative viscoelastic properties. Specifically, we demonstrate a phase-lag equivalence with probe-dependent active microrheology in a wide range of physically different, yet chemically identical materials. We exemplify the utility of Rheo-FLUCS in three distinctly different biological systems: compound-treated mouse fibroblasts (NIH-3T3), genetically modified human osteoblasts (U2OS) to elucidate the role of myosins in cytoplasmic mechanics, and early ascidian oocytes of *Phallusia mammillata* at fertilization stage. Our biological use-cases exemplify the application versatility of Rheo-FLUCS, which in the future may use phase information as a marker for developmental success.

Significance statement We present active and probe-free microrheology that introduces active measurements of relative viscoelastic information without the need for probe internalization. By establishing equivalence between flow- and force-based phase angles across diverse materials, including liquids, viscoelastic fluids, poroelastic gels and solids, we enable canonical rheological measurements in living systems, ranging from single cells to early embryos. This methodology circumvents the limitations of traditional techniques that rely on force probes as perturbative stimuli, which opens the door for a variety of use cases in biology that we demonstrate (including cells under pharmacological treatment, precision genetic mutant cell lines, and embryonic research). Although Rheological focused light-induced cytoplasmic streaming (Rheo-FLUCS) primarily reports viscoelastic changes within samples rather than absolute mechanical information, our findings bridge gaps in current methodologies and pave the way for probe-free and easy-to-use rheology as a predictive tool for physiological changes and disease progression.

## Introduction

Important roles of mechanics become increasingly recognized in the life sciences, and specifically in cell and developmental biology across scales, ranging from force-generating molecules (1, 2), through condensates with functional material properties (3–5), to ooplasm and embryonic mechanics (6–8). Passive rheological measurements may be probe-free but are known to be insufficient in measuring the mechanics of many out-of-equilibrium systems and active matter, such as living systems (9). The limited thermal motion of a probe poses an operational limit to the attainable viscoelastic range, and the generalized Stokes–Einstein relation, which bridges particle dynamics with material properties, breaks down (10). Active measurements, on the other hand, are capable of yielding faithful results even within living cells, but usually require the internalization of a mechanical probe and ensuring operation in the linear viscoelastic regime (11–13), which might be difficult to ascertain or report nonphysiological responses. Active measurements that are probe-free and of well-defined amplitude would therefore combine the advantages of two disparate approaches.

Previously, it has been suggested that thermoviscous flows (14, 15), the physical mechanism underlying focused light-induced cytoplasmic streaming (FLUCS) (16–21) can be used to assess relative mechanical properties of the cytoplasm by quantifying the flow displacement upon application of a driving stimulus (18). However, it remained to be shown that thermoviscous flows can also be used to extract canonical rheological parameters, such as the phase angle, as a quantitative metric of relative viscoelastic changes in complex, non-Newtonian fluids on the microscale (22–27). Leveraging the optical control capabilities of FLUCS, we advance the flow perturbations, yielding a new phase-sensitive microrheology method called Rheological focused light-induced cytoplasmic streaming (Rheo-FLUCS), which provides access to intracellular rheological changes in an active, yet probe-free way, thereby significantly advancing the fields of materials science and mechanobiology.

## Results

### Rheo-FLUCS: phase-sensitive active microrheology enabled by thermoviscous flows

Previously, it has been demonstrated how the rapid scanning of an infrared laser beam resulted in localized and directed flows within fluids of arbitrary viscosity, including the cytoplasm of a worm embryo or that of yeast cells (16, 18, 26, 27). These thermoviscous flows arise due to the combined effect of temperature-induced viscosity changes and thermal expansion of the fluids (14, 15). Periodically reversing the direction of laser scanning generates oscillatory flows, the amplitude of which was used to determine relative mobility of the cytoplasm as a proxy for its rheology (16).

Here, we conceptually extended the measurements to collect temporally resolved data, specifically from measurements that are highly synchronized with the laser-induced stimulus. This allows quantitative signal processing and access to phase-sensitive complex amplitudes of the oscillation, after appropriate Fourier-domain filtering.

In classic bulk rheology (28–30), stress–strain relations are widely used to characterize viscoelastic materials. For this, a periodic external force is used to induce oscillating stresses with angular frequency *ω*, which in turn produce an oscillatory shear deformation, with the same frequency but a temporal delay called “phase lag.” Stress that is strictly in-phase with the strain (phase angle $\varphi \;∼\;0^{\circ }$) signifies a purely elastic Hookean solid with finite frequency-dependent storage modulus $G^{\prime }(\omega )$ and comparably negligible frequency-dependent loss modulus $G^{\prime \prime }(\omega )$. On the other hand, a fully out-of-phase stress–strain relation ($\varphi \;∼\;90^{\circ }$) corresponds to a purely viscous Newtonian liquid with finite frequency-dependent loss modulus $G^{\prime \prime }(\omega )$ and negligible storage modulus $G^{\prime }(\omega )$. In-between these two extremes, the entire complex viscoelastic regime for non-Newtonian fluids is marked by phase angles $0^{\circ }<\varphi <90^{\circ }$, and specifically the relation $\tan\;(\varphi )=\frac{G^{\prime \prime }(\omega )}{G^{\prime }(\omega )}$ is frequently used to practically access the phase angle, as a relative measure of viscous and elastic properties of a material.

**Figure 1:**
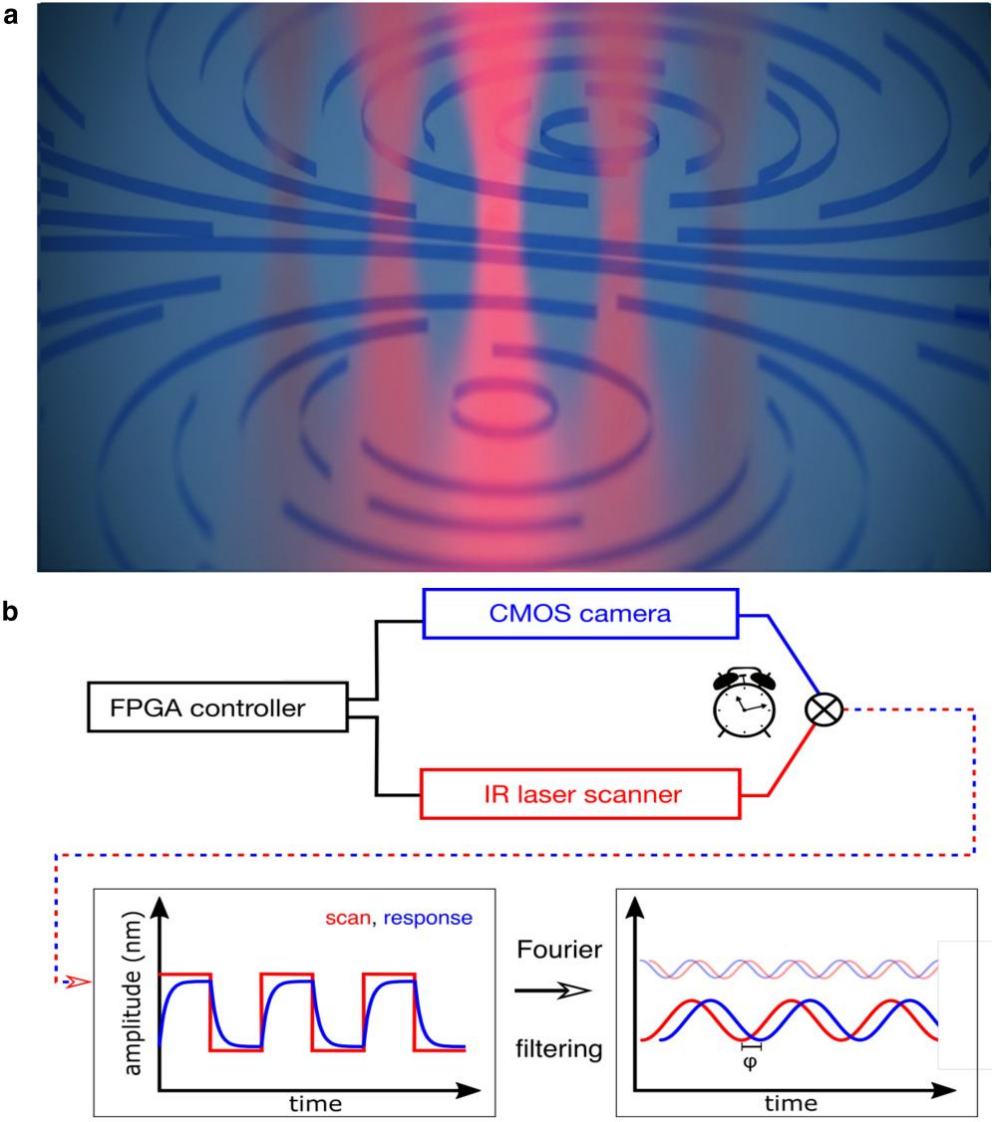
Conceptual and technical drawings of the phase-sensitive microrheology pipeline used by Rheo-FLUCS to extract rheological properties. a) Conceptual representation of the laser scanning (flow stimulus) as active oscillatory driving and the resulting time-delayed light-induced flows reported by an arbitrary feature in the image (eg dispersed nanoparticles in the fluid). In the oscillation description, brighter shades represent more recently sampled positions of the laser, whereas fainter shades indicate previously visited positions. b) Technical implementation of the microrheology pipeline that relies on synchronous triggering of the camera and the laser by a central controller. Utilizing this fine synchronization, we attributed any phase lag between the driving stimulus and the sample response to the material properties of the sample. This allows dynamic tracking of mechanical information in real time, where Fourier filtering eliminates passive and active noise present at all frequencies other than the excitation. FPGA, field programmable gate arrays; CMOS, complementary metal-oxide semiconductor.

In this work, we suggest that rather than requiring the use of an internalized force probe (31–36) to induce stresses that strain a material, flows that deform a medium can directly be induced via thermoviscous laser actuation, while still maintaining the quantitative phase relation between the driving stimulus and the material response (Fig. 1a). To this end, we directionally and repeatedly translate an infrared laser spot across the sample with a frequency in the low-kilohertz range, thereby inducing thermoviscous flows within it. The straining caused by this localized stimulus is monitored through the motion of a discernible feature within the sample, eg a tracer particle, which upon periodic switching of the scan direction reports on the sample response in the form of an observable oscillation (Fig. 1b).

**Figure 2:**
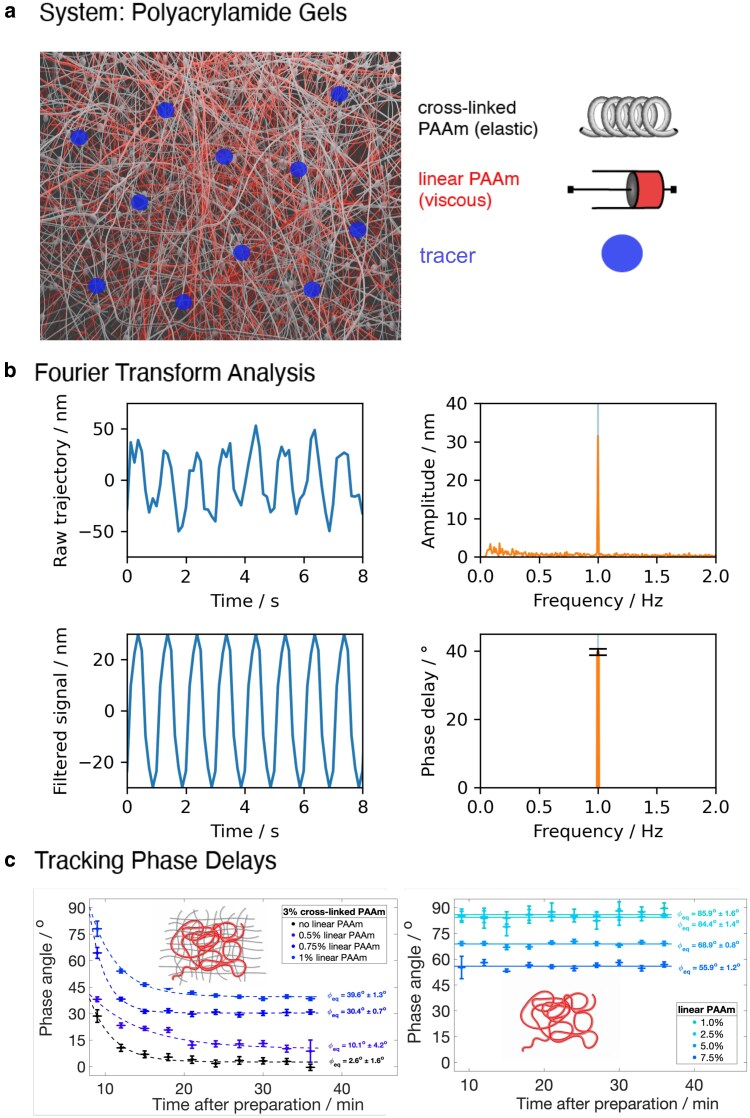
Viscoelastic model system, data extraction pipeline and sensitivity to the time-resolved solidification of fluids. a) Schematic representation of tunable PAAm gels employed as model viscoelastic systems: independent tuning of the elastic and viscous contributions is achieved through varying the concentrations of cross-linked (gray) and linear (red) PAAm, respectively. Oscillations are flow-induced, independently of mechanical probes. Small tracers merely report on the oscillations and are not required in systems, where resolvable image features already enable tracking. In this study, we used 1-µm carboxylated polystyrene particles due to the full transparency of the gels. b) Fourier filtering analysis is used to convert a noisy raw trajectory into low-noise canonical response of the material, where the clean signal is reconstructed by filtering for a given oscillation frequency. The laser driving (implicit stressing) begins at the start of the acquisition (reference time 0). This reconstructed signal provides access to the phase delay between the driving stimulus and the sample response, which reveals the mechanical properties of the test sample (data show 3 vol% cross-linked PAAm and 1 vol% linear PAAm probed at 1 Hz). The uncertainty in the phase lag originates from the relative magnitude of the amplitude compared to the Brownian noise floor. c) Real-time tracking of gelation in cross-linked PAAm gels (left) and entanglement in liquid suspensions of linear polyacrylamide chains (right). The dashed curves represent exponential fits with matching relaxation times for gelation (mixtures of cross-linked and linear PAAm), while solid curves confirm the relatively constant repeated measurements in viscoelastic fluids. Each data point of the same color represents a separate successive measurement on the same system. The errors in the individual phase angles were estimated based on the amplitude of each oscillation compared to a Brownian noise floor. The errors in the mean phase angles were estimated based on the standard deviation around the plateau (steady-state) region in each curve.

We argue and show that despite using an alternative stressor to conventional rheology, the phase angle, as canonical quantifier of fluidity, remains the same. We validate this hypothesis across a wide range of physically different, yet chemically identical systems established by Charrier et al. (37) (Fig. 2a). These allow the independent tuning of elastic ($G^{\prime }(\omega )$) and viscous ($G^{\prime \prime }(\omega )$) contributions in well-characterized environments.

### Real-time tracking of mechanical properties in model viscoelastic systems

First, we asked if Rheo-FLUCS can quantitatively measure the mechanical behavior of Newtonian liquids and report their chemically induced solidification. For this, we started by preparing dilute (highly fluid) linear polyacrylamide (PAAm) suspensions and oscillated 1-µm fluorescent polystyrene tracer beads to visualize the straining. Using a custom developed Fourier transform decomposition tool (Fig. 2b), we repeatedly extracted the same phase angle (Fig. 2c), with a value of φ around 85.91° ± 1.59°, close to a perfect liquid with virtually no elastic component.

Next, we investigated if the method could reliably report the real-time formation of a cross-linked gel (Fig. 2c, black data, 3 vol% PAAm, with monomer-to-bis-acrylamide cross-linker ratio of 100:1). The initial value of φ close to 30° signified the onset of gelation and we observed a progressive drop in the phase angle until a steady state was reached 20 min after sample preparation at around 2.63° ± 1.55°. These results so far show that Rheo-FLUCS is able to qualitatively report the dynamic solidification of a fluid, with a terminal phase angle characteristic of Hookean, perfectly elastic solids.

### Quantitative validation of phase angles using a force-driven approach

To confirm the equivalence between phase angles extracted from flow- and force-driven microrheology experiments, we compared a range of physically distinct, yet chemically nearly identical materials in their responses to both types of rheology. Specifically, in the force-driven microrheology we measured oscillatory step-stress perturbations via a magnetic force probe (Fig. S5 and Video S8).

**Figure 3:**
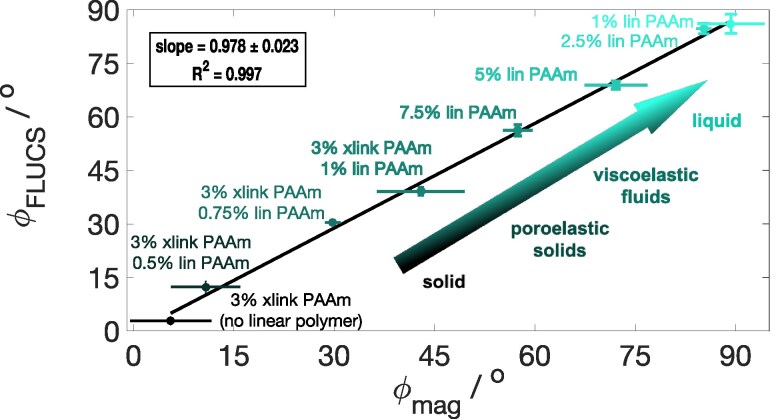
Equivalence of phase angles extracted via Rheo-FLUCS and force-driven rheology at 1 Hz. Direct comparison between phase lags obtained from Rheo-FLUCS and magnetic force driving for chemically identical, yet physically different mixtures of cross-linked (xlink) and linear (lin) PAAm, described in Fig. 2a. The data points represent weighted averages of five independent measurements, where the weighting was applied in relation to the fractional error in each phase angle. The linear fit confirmed the hypothesis that the stress–strain formalism relating phases and viscoelasticity from conventional bulk rheology offers a valid analytical treatment of data generated optofluidically with Rheo-FLUCS, where one measures within the linear viscoelastic regime.

To start, these measurements confirm that for both extremes, viz*.,* the predominantly fluid PAAm monomers and solidified PAAm gels, Rheo-FLUCS yields also quantitatively the same relative mechanical response as force-driven microrheology (Fig. 3).

Next, we assessed if also in the intermediate regime of viscoelastic materials, a direct linear proportionality for flow- and force-based measurements holds true. For this, we polymerized PAAm monomers into linear chains. In the absence of cross-linkers, this yields highly viscous solutions, with elasticity increasing with monomer concentration (from 2.5 vol% to 7.5 vol%) and phase angles decreasing down to about 60°, in agreement with the force-based reference measurements. The obtained relative material response can be attributed to polymer chain entanglement and storage of energy in the contacts.

As intracellular rheological properties might not only stem from viscous solutes or rigid filaments alone but also from the interaction of the cytosol with network elements of the cytoskeleton, we asked if Rheo-FLUCS is capable of accurately measuring phase angles of poroelastic materials. For this, we introduced increasing fractions of prepolymerized linear PAAm chains into the porous network of a cross-linked PAAm gel. While usually higher overall acrylamide concentrations have the tendency to form stiffer and more solid gels, the inclusion of linear chains into the network increases the viscous character of the gel due to strong frictional interaction between the solvent and the gel matrix (37). In these experiments, we find that the fluidity significantly increases due to the effective lubrication properties of the prepolymerized linear acrylamide that can equally be measured by Rheo-FLUCS.

Figure 3 presents a master curve, combining relative material information at 1 Hz over the entire viscoelastic range spanned by the model PAAm systems. Quantitatively, we observed identical phase angles in these physically different systems across the entire viscoelastic range of phase angles, from 0° to 90° ($R^{2}∼0.997$, when fitting the means of repeated experiments for each PAAm mixture). Moreover, we found the uncertainty via Rheo-FLUCS to be lower than that in the magnetic reference measurements, which in parts is likely due to the reduced drift of tracer particles in Rheo-FLUCS compared to the motion of force-subjected probes.

It is important to note here that our aim was not to perform complete material classification, which would otherwise require frequency-resolved measurements. Instead, the obtained validation, strictly holding true only for the selected frequency of excitation (1 Hz), serves to prove our previous phase-equivalence hypothesis between force-driven and flow-driven microrheology, at the same time confirming that both measurements fall within the linear viscoelastic regime.

Ensuring the latter linearity condition is particularly important in the case of probing soft biological systems, such as living cells, cytoplasm and active biological matter. In Rheo-FLUCS, we use nonforce-based perturbations with no explicit quantification of the stress amplitude. However, biological applications require small temperature changes, which prompt the need to operate at low laser powers, where linear relationships for fluid density and viscosity changes remain valid and only laminar flows are induced. Increased laser powers are generally expected to lead to a higher degree of straining and therefore the amplitude of oscillations alone cannot be used as a direct measure of the sample mechanics due to its dependence on operational parameters. Nevertheless, we extract the same response from our systems upon different laser powers (within calculated uncertainties), which may be seen as equivalent to performing a strain sweep in classical bulk rheology and observing strain-independent values for the storage and loss moduli. This attests to the linear character of the induced flows and confirms that we remain within the linear viscoelastic regime. Finally, to ensure a clear separation of timescales and absence of any nonlinear effects due to the time evolution of biological systems, in the following we use Rheo-FLUCS to probe each system at a reduced acquisition duration in comparison to the validation experiments above.

### Noninvasive intracellular rheology and sensitivity to drug treatment

**Figure 4:**
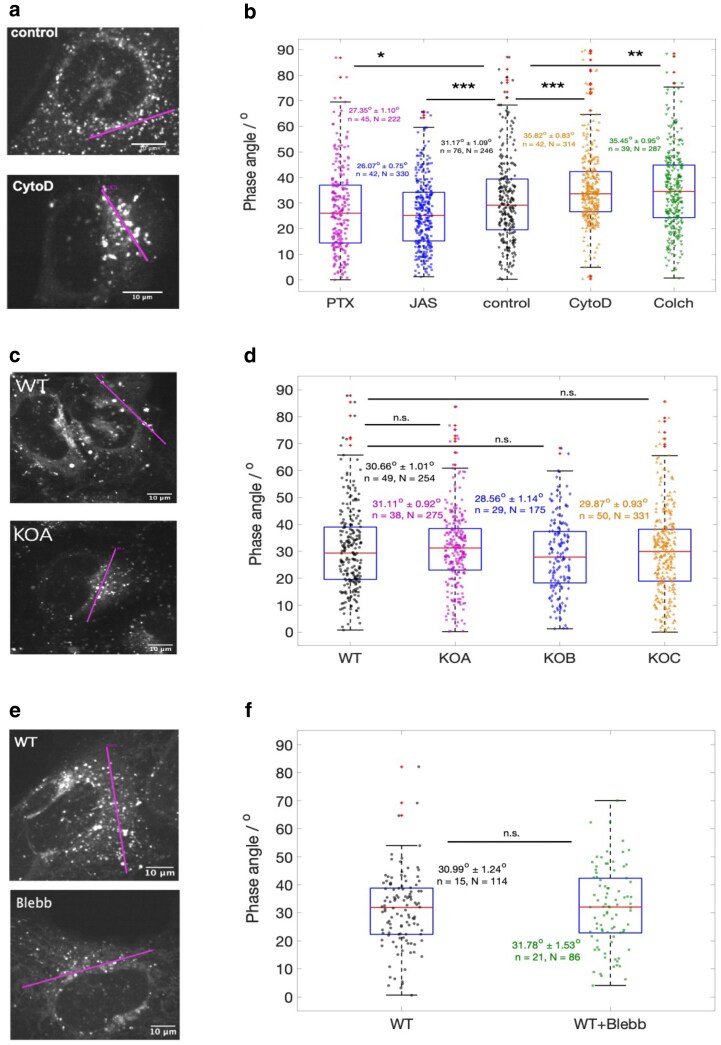
Active, probe-free microrheology in compound-treated mouse fibroblasts (NIH-3T3, a and b), precision knockout of NM II isoforms in mammalian osteoblasts (U2OS, c and d) and Blebbistatin-treated mammalian osteoblasts (U2OS, e and f). a) Fluorescent micrographs of WT and drug-treated NIH-3T3 cells (scale bars = 10 µm). The Rheo-FLUCS flows were applied along the magenta lines, where the exact scan location was varied for each independent experiment (each different cell), thus extracting information from different parts of the cytosol and averaging that information. LysoView 488 was used to stain the lysosome tracers. b) Box plot of phase angles measured via Rheo-FLUCS at 1 Hz. Lysosomes labeled with LysoView 488 served as tracers. In each case, we reported the weighted mean with its SE, number of cells (“*n*”), and number of oscillated lysosomes (“*N*”) from which information was extracted. In total, we analyzed 76 control cells in DMSO, 45 Taxol-treated (1 µM PTX) cells, 42 Jasplakinolide-treated (1 µM JAS) cells, 42 cytochalasin-D-treated (1 µM CytoD) cells, and 39 Colchicine-treated (2.5 µM Colch) cells, where treatment was applied 30 min prior to the acquisitions. All results were statistically significant when compared with the control (* for *P* < 0.05, ** for *P* < 0.01, *** for *P* < 0.001 as extracted from a two-sample t test comparing control and compound-treated cells). Red crosses mark outliers. c) Fluorescent micrographs of WT and genetically modified (knockout of NM IIA) U2OS cells (scale bars = 10 µm). The Rheo-FLUCS flows were applied along the magenta lines, where the exact scan location was varied for each independent experiment (each different cell), thus eliminating bias with respect to mechanical environment of certain parts of the cytosol and considering heterogeneity. LysoView 488 was used to stain the lysosome tracers. d) Box plot of phase angles measured via Rheo-FLUCS at 1 Hz. As with NIH-3T3, lysosomes labeled with LysoView 488 served as tracers. In total, we analyzed 49 WT osteoblasts, 38 cells with NM IIA knockout (KOA), 29 cells with NM IIB knockout (KOB), and 50 cells with NM IIC knockout (KOC). No statistical significance (“n.s.”) was found on applying two-sample t tests comparing the WT with each knockout individually. Red crosses mark outliers. e) Fluorescent micrographs of WT and Blebbistatin-treated (50 µM Blebb) U2OS cells (scale bars = 10 µm). The Rheo-FLUCS flows were applied along the magenta lines, where the exact scan location was varied for each independent experiment (each different cell) to present ensemble-averaged mechanical information of the cytosol environment. LysoView 488 was used to stain the lysosome tracers. f) Box plot of phase angles measured via Rheo-FLUCS at 1 Hz. In total, we analyzed 15 WT and 21 drug-treated osteoblasts. No statistical significance (“n.s.”) was found after applying a two-sample t test comparing the WT with Blebbistatin-treated cells. Red crosses mark outliers.

To test the applicability of Rheo-FLUCS in living cells and its potential as a probe-free technique, we started by using common NIH-3T3 adherent fibroblasts, in which we labeled lysosomes with LysoView 488 as fluorescent tracers (Fig. 4a). We find that by labeling these endogenous tracers (tens to hundreds per cell), we bring the sensitivity down to 30 nm oscillation amplitude per lysosome, resulting in phase noise in the range of 10° at 1 Hz oscillation frequency. Moreover, the simultaneously applied oscillations enable multiplexed readout, which in spite of the large heterogeneity, ensures high statistics.

Next, we asked if the method is suitable to detect changes in cell rheology, for which we used wild-type (WT) fibroblasts as well as cells treated with compounds known to mechanically stiffen or soften the cell interior. Specifically, we applied drugs employed in chemotherapy that cause alterations in the actomyosin cytoskeleton. This served as a positive control for probing differential mechanics.

First, we applied Paclitaxel (1 µM for 30 min) that is known to stabilize microtubules to the extent of preventing mitosis (38, 39). In these compound-treated NIH-3T3 cells, we measured lower phase angles relative to the WT, consistent with reduced cytosol fluidity (Fig. 4b). We obtained similar results on treating the mouse fibroblasts with Jasplakinolide (1 µM for 30 min), which was previously reported to promote actin polymerization in vitro, leading to stiffening of the cytoplasm through strengthening the cytoskeleton (40, 41). These findings are in agreement with previous reports from microrheology based on atomic force microscopy, where a predominantly elastic response and relative increase in stiffness were observed by Laudadio et al. (42) upon treating rat airway smooth muscle cells with Jasplakinolide.

In contrast, we found upon applying Colchicine (2.5 µM) that suppressing polymerization of microtubules leads to fluidization of the cytoplasm, as indicated by the higher phase angles in Fig. 4b (43, 44). Similar effect to the cytosol mechanics we measured upon applying Cytochalasin D (1 µM), which can be attributed to capped actin filaments that prevent further polymerization and, as with Colchicine, effectively weakening the cytoskeleton (45–47).

We conclude from these measurements that Rheo-FLUCS is capable of detecting changes in adherent cells as small as 4° in the phase angle at 1 Hz frequency and with uncertainty of ca. 1°. This is comparable to the difference observed between 1 vol% linear PAAm in water and pure water. Despite the sample-based variability, the multiplexing yields powerful statistics.

### Osteoblast cytoplasm mechanics is insensitive to myosin activity

Motivated by recent findings obtained with a cell microstretcher (48), showing that cortex mechanics is finely controlled through a complementary interplay of three nonmuscle myosin II (NM II) isoforms (A, B, and C), we asked if the mechanical properties of the cytoplasm are similarly dependent on motor activity as the cortex. Previously, it was suggested that NM IIA is essential for building up cellular tension during initial stages of force generation, whereas NM IIB is required to elastically stabilize NM IIA-generated tension. On the other hand, a new role of NM IIC in establishing tensional homeostasis has been revealed (48).

To investigate further the individual contributions of each NM II isoform to cytosol mechanics, we performed NM IIA, NM IIB, and NM IIC CRISPR/Cas-based genetic knockouts in U2OS mammalian osteoblasts (49, 50). In each case we did not detect any observable change compared to the WT (Fig. 4c and d), thereby concluding that, although NM II isoforms play a major role in establishing a fine balance of mechanical forces in the cortex, individually they do not significantly contribute to cytosol stiffness.

As none of the motor knockouts led to substantial changes of cytoplasm mechanics individually, we asked if NM isoforms could still have redundant functions with respect to cytoplasm mechanics. Therefore, we applied a compound treatment (50 µM Blebbistatin, Fig. 4e and f) in the same mammalian U2OS cell line. Applying Blebbistatin drug treatment to suppress all motor activity simultaneously resulted in no net effect on cytosol mechanics, consistent with recent measurements in isolated zebrafish cells using optical tweezers (51) and emphasizing that the isoforms neither have individual roles with regards to cytoplasm mechanics, nor do they have redundant roles. This observation was further corroborated by applying 50 µM Blebbistatin treatment in NIH-3T3 cell line (Fig. S1).

Comparing with the work of Weißenbruch et al. (48), we note a disparate response in cytosol and cortical mechanics, which seems unexpected since the actomyosin cytoskeleton has been identified as the canonical regulator of cell shape. Rapid cell changes induced by anisotropic cortical contractions have a profound impact on the cytosolic fluid dynamics during cell migration and cytokinesis (52–54). However, although inhibition of actomyosin contractility was shown to reverse cytosolic flow direction in keratocytes (52), our results suggest that cortical rigidity does not intrinsically influence the stiffness of the cytosol in adherent mesenchymal cells.

The Rheo-FLUCS results emphasize the importance of deciphering the mechanisms responsible for cortex-cytosol mechanical interplay that appear to reach beyond a simple description of actomyosin cytoskeleton-membrane coupling.

### Applying FLUCS microrheology to ascidian oocytes

Ooplasm mechanics play a pivotal role in development within biological systems and hold significant promise for applications in reproductive medicine (55–57). For example, in mouse oocytes, asymmetric streaming crucially positions the spindle complex, underscoring the intricate choreography involved in embryonic development (58, 59). Moreover, friction forces are instrumental in dictating cytoplasmic reorganization, positioning material and inducing shape changes in ascidian oocytes upon fertilization (6). Given the contribution of the mechanical properties of oocytes to embryonic development, we asked whether we could measure using Rheo-FLUCS ooplasmic properties of ascidians, a simple yet powerful model organism of the large phylum *Chordata*, with an egg diameter of around 150 µm.

**Figure 5:**
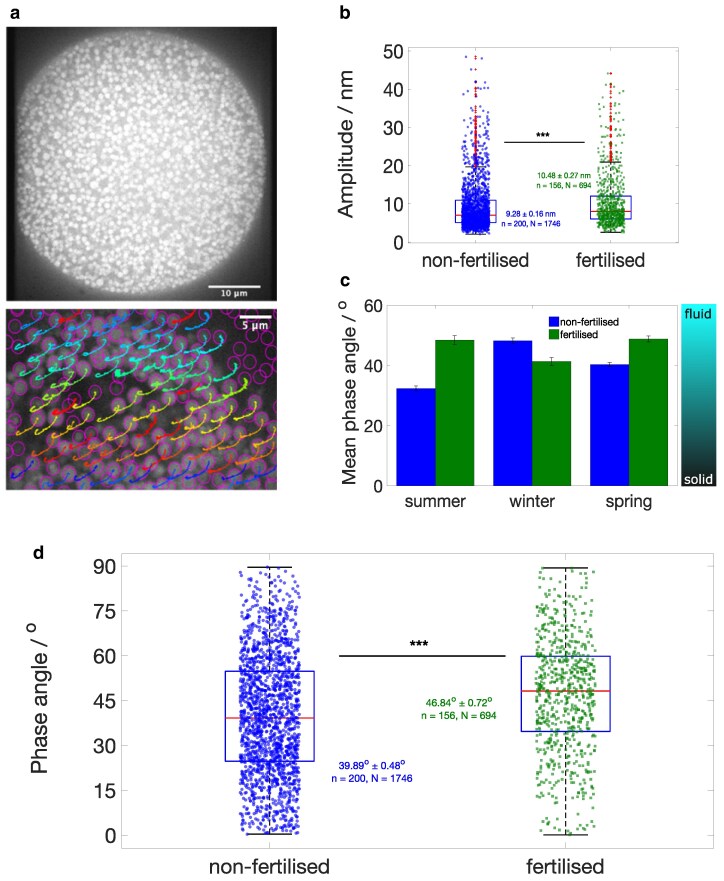
Mechanosensing in nonfertilized and fertilized ascidian oocytes (*P. mammillata*). a) SYTO-59 labeling of yolk granules within a dechorionated ascidian oocyte (scale bar = 10 µm). The yolk granules were simultaneously oscillated via Rheo-FLUCS at 1 Hz frequency and then tracked via the TrackMate plugin on Fiji (scale bar = 5 µm). This enabled high-throughput measurements. b) Amplitude box plot generated from the oscillatory motion of yolk granules in nonfertilized and fertilized (first-polar-body stage) ascidian eggs. Despite finding an increased amplitude in fertilized eggs, we could not conclusively link that amplitude to changes in ooplasmic mechanics, due to the dependence of the amplitude on experimental parameters, such as location of the scan path with respect to the oscillated feature and power of the laser. c) Mean phase-angle data collected across three different seasons, displaying variations in the mechanical response of both nonfertilized and fertilized oocytes. Each error bar represents SEM, where the data were collected from a total of 20 nonfertilized and 15 fertilized eggs in the summer, 65 nonfertilized and 45 fertilized eggs in the winter, and 115 nonfertilized and 96 fertilized eggs in the spring. The color bar accompanies the y-axis values and facilitates the interpretation of the phase angle (low values correspond to solid-like environment, and high values to liquid-like medium). d) Phase-angle box plots, showing the overall increase in phase angle upon fertilization across all three seasons. As in (b), we report the distribution's mean and SEM, number of analyzed oocytes (“*n*”), and number of oscillated yolk granules (“*N*”). The differences in both cases were found to be statistically significant (*P* < 0.001) on performing a two-sample t test. These proof-of-principle measurements were of limited duration due to challenges associated with photobleaching and constitute the basis for ongoing work that aims to determine seasonal variations in ooplasm fluidity.

To achieve noninvasive measurements, we labeled yolk granules in *Phallusia mammillata* oocytes with SYTO 59 and employed Rheo-FLUCS to oscillate them instead of microbeads (Figs. 5a, S2–S4). Due to the high density of yolk granules, these measurements could be highly parallelized. By targeting thousands of yolk granules per confocal section in each oocyte, we achieved comprehensive mechanical profiling across multiple positions simultaneously.

In this work, we encountered several factors that influence ooplasm mechanics, contributing to better understanding of how these dynamics are regulated within ascidian oocytes. First, we analyzed the motion of the yolk granules and extracted the amplitudes (Fig. 5b) and phase angles (Fig. 5c and d) of oscillations induced by Rheo-FLUCS. We compared those before and shortly after fertilization. The reference measurement point in all fertilization experiments was the ejection of the first polar body during meiosis. Overall, fertilized oocytes exhibited increased fluidity compared to their nonfertilized counterparts. This finding aligns with existing reports indicating that active processes initiated by fertilization enhance the fluid dynamics of the ooplasm, thereby facilitating subsequent developmental stages (60–63). The increase in fluidity likely plays a crucial role in enabling cellular rearrangements necessary for successful embryonic progression.

Further, our research identified significant seasonal variations in ooplasm rheology (Videos S1–S6). These findings complement existing literature that highlights the seasonality associated with reproductive potential in various species (64–66). This highlights the promising use of Rheo-FLUCS in the field of reproductive medicine for predicting or evaluating reproductive capacity based on rheological properties. Ooplasm rheology could thus serve as a window into the intricate processes governing early development, with the outlook of advancing fertility treatments and improving conservation efforts aimed at preserving biodiversity.

## Discussion

In this work, we demonstrated that intracellular active rheology is possible without the need for an internalized force probe and in the presence of other nonequilibrium processes. By establishing the equivalence of flow- and force-based phase angles across a wide range of PAAm mixtures, with fundamentally different microscopic properties underlying their emergent rheological behaviors (liquids, viscoelastic fluids, poroelastic gels, and solid gels), we enable canonical rheological measurements in the linear viscoelastic regime even in living systems, from single cells to early embryos.

Like any method, Rheo-FLUCS also comes with certain limitations, which we acknowledge here: due to its active and probe-free nature, Rheo-FLUCS does not benefit from established conversion of particle dynamics into viscoelastic moduli, as otherwise enabled by equilibrium thermodynamics. The method currently delivers only relative information about stiffness, as defined by the ratio of *G*ʹʹ(*ω*) and *G*ʹ(*ω*). Furthermore, the phase angle we obtain is inherently frequency-dependent, and extraction of the complete viscoelastic spectrum of a material requires performing measurements over a broad range of frequencies. To this end, single-frequency measurements do not provide a full description of how material mechanics change.

However, Rheo-FLUCS clearly opens new avenues for exploration in materials science and mechanobiology, where direct access to mechanics was previously limited due to compartmentalization and force probe incompatibility. The contactless, laminar flows induced by Rheo-FLUCS both extend the measurable viscoelastic range of complex fluids to very highly viscous, pitch-like samples (Fig. S6 and Video S9) and enable readout of the linear mechanical state of cellular cytoplasm and embryonic ooplasm. Notably, our methodology delivers reliable mechanical data even in nonequilibrium and highly compartmentalized systems owing to phase-locked detection, which mitigates the influence of contributions at frequencies other than the frequency of excitation. Within this context, active fluctuations only affect uncertainty, and do not introduce any bias. Further, we perform measurements on the timescale of seconds, ensuring we remain in the linear viscoelastic regime, where the biological system under investigation does not change substantially. While Brillouin spectroscopy informs about mechanical properties at gigahertz frequencies (22–25), where thermal diffusion dominates, our technique extends noninvasive measurements to well below 10–100 Hz (Fig. S7 for measurements at multiple frequencies in a photoresist material for photolithography), where active processes are known to have an influence on the net mechanical properties (67). Successive phases of diffusion and active transport have been shown through temporal analysis of single-particle transport on trajectories of labeled vesicles (63, 68) or viruses (69) in single cells, where path dissection was performed manually (70). Hence, measuring cell mechanics on timescales longer than 10 ms necessitates the use of active methods discriminating between passive and active motion (71, 72).

Finally, this opens possibilities to use Rheo-FLUCS in the detection of physiological changes in cellular phenotypes, eg as was previously shown via the cell's metabolic state (16), and gain additional power and universality by expanding the readout into the time domain. Within the life sciences, performing Rheo-FLUCS could enable future uses of probe-free rheology as a diagnostic tool for disease and reproductive medicine (66), where advanced tracking schemes may remove the need for introducing any fluorescent labels (Videos S10 and S11).

## Methods

### FLUCS interactive microscopy setup

All experiments were conducted using the FLUCS microscope described in our previous work (20), where simultaneous external triggers were sent synchronously to the laser scanner (two-axis acousto-optic deflector, model AA.DTSXY-A6, Pegasus Optik) and the imaging device (Zyla 5.5 sCMOS camera). The infrared laser beam (1,455 nm wavelength, Raman laser, CRFL-20-1455-OM1, 20 W, Keopsys, CW mode) was focused through a custom-coated 60× objective lens (UPLSAPO, NA = 1.2, W-IR coating, Olympus) and was then used to generate dynamic heat patterns. The laser scanning in all microrheological experiments was performed at 2 kHz scan rate, where the laser was repeatedly scanned 1,000 times along each of two opposite directions. Since the direction of the induced flows is determined by the direction of laser scanning, the flow direction was reversed every 0.5 s. This effectively induced steady-shear oscillations (Video S7) with a period of close to 1 s, which contains an approximate error of 600 µs due to signal processing. The measured temperature increase in the range of laser powers used here is 1–3 K.

### Software control of the FLUCS setup

Custom-written LabView software was used to generate the waveforms required for scanning the laser via the acousto-optic deflector. A data acquisition card (cf., Mittasch et al. (16)) served as an analog-to-digital converter and enabled rapid signal transfer between the computer and the laser scanner. The frequency and transition of the waveforms were verified using an oscilloscope (Datatec, HMO72). The LabView software allowed real-time modifications to the frequency of laser scanning, linear extent of the flows, number of scan repetitions, duration of the acquisition and other experimental parameters.

### Software for analyzing active, phase-sensitive microrheology data

Imaging was performed with a Zyla 5.5 sCMOS camera and the motion of probe-particles tracked via Fiji's open-source TrackMate plugin (73). Subsequently, the tracked particle coordinates and temporal data were forwarded to an in-house Python routine that served as a monochromatic filter that selects viscoelastic data at the frequency of oscillation. Most notably, the routine allowed decomposition of the filtered signal into an amplitude and a phase, where the latter served as a precise measure of material properties. The routine required taking 2^n^ images due to the Fourier transform processing, which necessitated measurement acquisitions as long as 64 s (cells and embryos samples) or 128 s (nonliving, complex fluids). The generation of box plots and statistical analysis were performed on Matlab.

### Linear and cross-linked PAAm preparation

We followed the protocol for preparing PAAm gels outlined by Charrier et al. (37). Linear PAAm solutions (50 mL volume) were prepared in the absence of a cross-linker by mixing 40 vol% acrylamide (Bio-Rad, #1610140) with *N*, *N*, *N*ʹ, *N*ʹ-tetramethylethylenediamine (25 µL TEMED, Sigma-Aldrich, T9281) and 10 vol% ammonium persulfate (120 µL APS, Sigma-Aldrich, A3678). The mixture was then degassed using a vacuum desiccator for 30 min and incubated overnight at 37 °C. The resulting highly viscous solution was combined in different proportions with a cross-linked PAAm gel prepared using 40 vol% acrylamide, TEMED, APS and 2 vol% bis-acrylamide (Bio-Rad, #1610142). The final acrylamide (monomer-to-bis) ratio was 100:1.

The probe-particles we used in the flow-based microrheology experiments on PAAm gels were fluorescent (Dragon Green) 1,037-µm carboxylated polystyrene beads (PS-COOH uniform dyed microspheres, Bangs Laboratories, Inc., FC04F). These were diluted 10^3^× in the test sample and then pipetted into a 15-µm tall chamber composed of a plain microscope slide (76 × 26 × 1 mm, Paul Marienfeld GmbH & Co. KG, Germany) and a thin round cover slip (22-mm diameter, 170-µm thickness, borosilicate glass). The chambers were sealed with two-part impression material (Vinylsiloxanether, Identium, 13711) to minimize evaporation over the course of the measurements. The measurement duration in each case was around 128 s to generate 1,024 images, required for the Fourier transform analysis and sufficiently high signal-to-noise ratio.

### Magnetic microrheology validation measurements

For performing magnetic force-driven microrheology experiments, we used an electromagnetic needle as reported in Stoev et al. (20). The needle was of 4.5-mm diameter, 100-mm length and the core of the electromagnet was Hy-Mu 80 (80% nickel–iron–molybdenum alloy, rod dimensions of 0.380 × 0.135 in, National Electronic Alloys Inc., Oakland, NJ), enclosed within a brass frame. To ensure the induced magnetic forces were sufficiently strong to oscillate particles within PAAm gels, we modified the tip angle to 30° and the number of coil turns was increased to 650. We performed the experiments in an open-chamber configuration, where the sample was loaded onto a customized MatTek dish with a wall opening for the needle. The latter was connected to a laboratory DC power supply (EA Elektro Automatik, EA-PS 3016-10B) via an LED driver that allowed periodic on-off pulsing of the magnetic field. The probe-particles we employed for these measurements were fluorescent (Dragon Green) 0.9-µm magnetic polymer-coated beads (PS/6% Divinylbenzene/V-COOH Mag 480-520, Bangs Laboratories, Inc., MCDG001) and 8.26-µm magnetic COMPEL beads (COOH-modified, 480-520, Bangs Laboratories, Inc., UMDG003) to test for size-dependent effects. The measurement duration in each case was around 128 s to generate 1,024 images, required for the Fourier transform analysis and sufficiently high signal-to-noise ratio.

### Preparation of NIH-3T3 cells for FLUCS microrheology

Mouse embryonic fibroblasts (NIH-3T3) were cultured in DMEM high glucose (Gibco, 31966-021) supplemented with 10% fetal bovine serum (Gibco, 10270-105) and 1 µg/mL penicillin and streptomycin (Gibco, 15140-122) in a humidified incubator with 5% CO_2_ at 37 °C.

For the microrheology experiments, the cells were seeded onto a clean glass coverslip and allowed to reach ∼50% confluency. On the day of the FLUCS experiment, 30 min in advance the cells were treated either with DMSO or with compounds matching the concentrations, viz., 2.5 µM Colchicine (Sigma-Aldrich, C3915), 1 µM Paclitaxel (Sigma-Aldrich, T7191), 1 µM Jasplakinolide (Sigma-Aldrich, J4580), and 1 µM Cytochalasin D (Sigma-Aldrich, C8273), and costained with LysoView 488 (1:800 dilution, Biotium, 70067). After the treatment, the cells were carefully loaded onto an experimental chamber made of a thick sapphire glass slide with Peltier elements on each side to control the temperature, as described by Mittasch et al. (16). Polystyrene beads (15-µm diameter) dispersed 1,500× in culturing medium were added as a spacer between a cover slip and a microscope slide to prevent excessive compression of the cells during the experiments.

The FLUCS microrheology experiments were then conducted by generating linear oscillatory scans (1-Hz frequency) in the cytoplasm of the cells and away from the nucleus. The stained lysosomes were used as tracers and their motion subsequently analyzed using the same particle tracker and Python code as with the synthetic probe-particles in the PAAm gel experiments. Only phase lags with uncertainty <30° were included in the final plot. The measurement duration in each case was around 64 s to generate 512 images, required for the Fourier transform analysis and sufficiently high signal-to-noise ratio.

### Preparation of U2OS osteoblasts for FLUCS microrheology

Human osteosarcoma cell line (U2OS), WT, and CRISPR knockouts for the NM II isoforms A, B, and C genes, were used to determine the effect of each gene on tensional homeostasis. As with NIH-3T3, U2OS cell lines were cultured in DMEM (Gibco, 31966-021) supplemented with 10% FBS (Gibco, 10270-105), 1 µg/mL Penicillin streptomycin (Gibco, 15140-122). The cells were maintained in a humidified 5% CO_2_ incubator at 37 °C. For the Rheo-FLUCS experiments, the cells were seeded onto a glass cover slip to reach 50–60% confluency. On the day of the experiment, the cells were treated with LysoView 488 (1:800 dilution, Biotium, 70067) for 30 min. The cover slips were then carefully loaded onto an experimental chamber made of a thick sapphire glass slide with Peltier elements on each side to control the temperature, as described by Mittasch et al. (16). Polystyrene beads (15-µm diameter) dispersed 1,500× in culturing media were added as a spacer between a cover slip and a microscope slide to prevent excessive compression of the cells and preserve the viability of the cells over the course of the experiments. The measurement duration in each case was around 64 s to generate 512 images, required for the Fourier transform analysis and sufficiently high signal-to-noise ratio.

### Preparation of ascidian oocytes for FLUCS microrheology

*Phallusia mammillata* were obtained from Roscoff Marine Station (EMBRC, France) and kept in artificial seawater (ASW; 36 g/L Tropic Marin BIO-ACTIF Sea Salt, Tropic Marine) at 16 °C for 3–4 weeks under constant light. Eggs and sperm were harvested and kept at 4 °C for 3–4 days. Eggs were dechorionated with 1% trypsin (Sigma-Aldrich, T8003) in ASW for 40 min under gentle agitation and kept in ASW supplemented with 0.05 g/L streptomycin sulfate salt (Sigma-Aldrich, S6501). Dechorionated and nonfertilized eggs were incubated with 1 µM SYTO 59 red fluorescent nucleic acid stain (Invitrogen, S11341) in ASW for 5 min and washed with ASW prior to fertilization. Ascidian sperm was activated with pH 9 ASW and used to fertilize dechorionated eggs. All imaging and FLUCS experiments were conducted at 15 °C.

We performed Rheo-FLUCS experiments and analysis as with the NIH-3T3 cells, where in the ascidian eggs we generated linear oscillatory scans deep into the ooplasm and away from the cell membrane. These were again conducted at 1-Hz oscillation frequency, at which we compared the response of nonfertilized and fertilized eggs. The measurement duration in each case was around 64 s to generate 512 images, required for the Fourier transform analysis and sufficiently high signal-to-noise ratio.

### Statistical analysis of data in box plots

In the measurements with all biological systems, we have used box plots, including only phase-angle data within the physically meaningful region of 0° (perfect elastic solid) to 90° (perfect Newtonian liquid), and uncertainty <30°. The fractional uncertainties in the phases were estimated from the fractional uncertainties in the oscillation amplitudes (ratio of signal-to-noise), where proportionally higher weighting was assigned to phases with lower uncertainty. This contributed to performing weighted two-sample t tests, where the control or WT was compared to each treatment or knockout individually. Statistical significance was then assigned based on the *P*-value (n.s. for *P* > 0.05, * for *P* < 0.05, ** for *P* < 0.01, *** for *P* < 0.001) in the two-sample t test.

The box plots indicated median values and interquartile range, with 75th and 25th percentiles forming the boundaries of the box. The whiskers projected out to the extreme, statistically significant values within the dataset, while red crosses signified outliers.

## Supplementary Material

pgag190_Supplementary_Data

## Data Availability

All data and materials are made available on Zenodo public repository under Creative Commons Attribution 4.0 International License: https://doi.org/10.5281/zenodo.20322020.

## Preprints

This manuscript was posted as a preprint: https://doi.org/10.1101/2025.04.07.647540.
